# The potential hematological features of children with spastic cerebral palsy in China: a retrospective multidimensional data analysis based on routine hematological indicators

**DOI:** 10.3389/fped.2026.1765309

**Published:** 2026-03-24

**Authors:** Yanjun Mo, Yu Jiang, Zhaozhan Qiang, Gang Liu, Ruiqin Yu, Huizhong Bai, Ying Zeng, Chuangyu Hu, Jiashu Yue, Zhuoluo Zhou, Jingpei Ren, Lin Xu, Xiaoye Li, Xiaohong Mu

**Affiliations:** 1 Dongzhimen Hospital, Beijing University of Chinese Medicine, Beijing, China; 2 Hubei University of Chinese Medicine, Wuhan, China; 3 Xi'an Children’s Hospital, Xi'an, China

**Keywords:** biomarkers, children, hematological features, retrospective study, spastic cerebral palsy

## Abstract

**Background:**

Cerebral palsy (CP) is a non-progressive brain injury primarily characterized by abnormal posture and movement disorders. Among them, spastic cerebral palsy (SCP) accounts for 70% of cases. Previous small sample hematological data analyses have revealed significant differences in inflammatory marker ratios between SCP patients and healthy controls. This study aims to expand the sample size and perform a multidimensional data analysis using routine hematological indicators to identify hematological features of spastic cerebral palsy, potentially providing new directions for the treatment of SCP.

**Methods:**

This retrospective study included 305 children with spastic cerebral palsy and 149 healthy children, aged 3–12 years. Previous routine blood and biochemical test results were collected from the participants. Statistical analysis was performed on clinically common indicators and related composite indicators, and subgroup analyses were conducted based on age group (preschool vs. school-age).

**Results:**

Compared to the healthy control group, SCP patients had significantly lower levels of NPAR, alkaline phosphatase (ALP), creatinine (Cr), SII, and MPV/PC (*p* < 0.05). AST/ALT, NLR, total protein, and SIRI levels were significantly higher in the SCP group (*p* < 0.05). Logistic regression analysis showed that ALP, Cr, SII, and MPV/PC were protective factors for SCP, while AST/ALT and NLR were risk factors for SCP. Combining these indicators for SCP diagnosis, the ROC curve analysis yielded an AUC of 0.781. Subgroup analysis showed that children aged 3–6 years with SCP had significantly lower Cr, AST/ALT, SII, MPV/PC, and NLR levels compared to children aged 7–12 years with SCP. Furthermore, creatinine, AST/ALT, SII, MPV/PC, and NLR levels were positively correlated with the age of SCP children.

**Conclusion:**

This study reveals a significant association between alkaline phosphatase, creatinine, AST/ALT, SII, MPV/PC, NLR in routine blood indicators and the risk of SCP, providing important reference for clinicians to monitor the health status of children with cerebral palsy, optimize treatment plans, and implement nutritional interventions.

## 1 Introduction

Cerebral palsy (CP) refers to a non-progressive brain injury caused by factors such as premature birth, difficult delivery, asphyxia, or jaundice. It is a lifelong physical disability affecting movement and posture. Motor impairments in CP are usually accompanied by sensory, perceptual, cognitive, communication, and behavioral disorders, as well as epilepsy and secondary musculoskeletal problems. In high-income countries, the incidence has decreased from 2.1 per 1,000 live births to 1.6, a reduction of 40%. However, the incidence remains high in low- and middle-income countries (1). Spastic cerebral palsy (SCP) accounts for about 70% of CP cases, characterized by increased muscle tone, hyperactive tendon reflexes, and muscle spasms, often leading to limb deformities, with severe cases resulting in the loss of normal walking ability.

Compared to healthy children, school-aged (3–12 years old) children with SCP face multiple health challenges during their growth and development. First, malnutrition is one of the most common complications in children with cerebral palsy. Due to motor impairments, feeding difficulties, gastrointestinal issues, and increased energy expenditure, 57.6%–80% of children with cerebral palsy experience malnutrition, which may manifest as anemia, hypoalbuminemia, and trace element deficiencies (2). Malnutrition affects the overall health, growth, and participation in education and social activities of children (3). Additionally, a large proportion of children with cerebral palsy have epilepsy, ranging from 15% to 60%. Some patients require long-term treatment with antiepileptic drugs (AEDs) (4), and various AEDs (such as valproate, phenytoin, etc.) may cause adverse reactions in the nervous system, hematologic system, and metabolic abnormalities. Furthermore, due to malnutrition and relatively low immunity, children with cerebral palsy are more prone to recurrent respiratory and lung infections (5). Chronic infections and sustained inflammatory states may lead to elevated inflammatory markers, worsened anemia, and changes in immune cell proportions. It is noteworthy that inflammation is not only a common complication in children with cerebral palsy but is also considered one of the important factors contributing to central nervous system damage in these children. One of the most significant underlying pathophysiological mechanisms of cerebral palsy is the inflammation and infection within the amniotic cavity, which triggers an inflammatory response and causes damage to the developing brain (6). The neurodevelopmental dysfunction observed in children with cerebral palsy is also related to immune activation and inflammation in early life (7). At the metabolic level, plasma energy metabolism, protein synthesis, and amino acid metabolism in children with spastic cerebral palsy differ from those in typically developing children (8). Among them, glutamate is the metabolite with the highest differential abundance in the brains of cerebral palsy patients, suggesting that glutamate may be a potential biomarker for cerebral palsy (9). Metabolic-related research also provides a foundation for studying the complex pathophysiology of cerebral palsy and monitoring disease progression.

However, in clinical practice, especially in resource-limited primary healthcare institutions, early identification and continuous monitoring of these potential health issues in children with cerebral palsy are often inadequate. Although magnetic resonance imaging, professional nutritional assessments, and complex immunological tests can provide important information, their accessibility is limited in low- and middle-income countries or remote areas. In contrast, routine blood tests and biochemical examinations, which are the most common and cost-effective diagnostic tools in primary healthcare settings, are widely used for child healthcare and disease screening (10). Peripheral inflammation and metabolic-nutritional status can be reflected to some extent through routine blood tests and biochemical indicators. Specifically, blood tests are primarily used to assess the patient's inflammation and immune status, while biochemical tests mainly reflect the body's metabolic function. This provides a basis for monitoring biomarkers of cerebral palsy from routine blood data. If the hematological parameter characteristics of school-aged children with spastic cerebral palsy can be clarified, it will help clinicians early identify malnutrition, potential infections, and metabolic abnormalities, thereby allowing for timely adjustment of nutritional support plans, optimization of treatment drug selection, or initiation of infection prevention and control measures, ultimately improving the overall health status and quality of life of the children. Therefore, exploring an auxiliary disease monitoring tool based on routine tests (such as blood routine and biochemistry) widely conducted in primary healthcare institutions holds significant clinical value and public health importance.

This study compares and describes the hematological parameters of a large sample of school-aged children with spastic cerebral palsy and healthy children, systematically analyzing the characteristics of hematological indicators in children with spastic cerebral palsy, providing scientific evidence for early clinical intervention and comprehensive management.

## 2 Methods

### 2.1 Study design

This retrospective study was conducted at Dongzhimen Hospital of Beijing University of Chinese Medicine and Xi'an Children's Hospital. A total of 305 children with cerebral palsy and 149 healthy controls were recruited between January 2018 and January 2025. The study was approved by the Clinical Research Ethics Committee of Dongzhimen Hospital, Beijing University of Chinese Medicine (No: 2024DZMEC-292-03).

**SCP Inclusion Criteria:** The diagnosis of spastic cerebral palsy was based on the definitions, classification, and diagnostic standards recommended in 《Cerebral Palsy: Modern Surgical Treatment and Rehabilitation》 (11). The age range of participants was 3–12 years.

**SCP Exclusion Criteria:** Other types of cerebral palsy; Presence of severe cardiovascular, pulmonary, liver, kidney, or other systemic diseases; Cerebral palsy caused by other factors such as infections or jaundice; Genetic, metabolic, neurodegenerative diseases, brain tumors, or brain injuries that cause cerebral palsy-like changes; Use of medications or treatments that may affect the study results (For example, immunosuppressants: methotrexate, azathioprine, etc., long-term systemic corticosteroids, anticoagulants: warfarin, heparin, etc., antiplatelet drugs, and recently started iron supplements, which are relevant medications that affect hematological parameters.); Presence of immunodeficiency, coagulation disorders, or other similar conditions.

**Inclusion and exclusion criteria for the healthy control group:** Inclusion criteria: Aged 3–12 years; normal neurodevelopment confirmed by clinical assessment; no abnormalities on physical examination. Exclusion criteria: Presence of serious internal diseases such as cardiac, pulmonary, hepatic, or renal disorders; Current use of medications or therapies that may affect the study results; Immunodeficiency, coagulation disorders, or other related conditions; History of acute infection or fever within the past 2 weeks.

### 2.2 Inclusion criteria indicators

**Participant Information:** Gender, age, and date of testing (year/month/day).

**Immune and Inflammatory Indicators:** White blood cell count (WBC), neutrophil count (NEU), lymphocyte count (LYM), monocyte count (MONO), neutrophil percentage (NE%), platelet count (PLT), red blood cell distribution width (RDW), mean platelet volume (MPV).

**Metabolic Indicators:** Total protein (TB), albumin (ALB), alanine aminotransferase (ALT), aspartate aminotransferase (AST), alkaline phosphatase (ALP), creatinine (Cr), blood urea, uric acid (UA).

**Composite Indicator Calculation Methods:**Platelet-to-Lymphocyte Ratio (PLR): Platelet count/Lymphocyte count; Systemic Immune-inflammation Index (SII): (Platelet count × Neutrophil count)/Lymphocyte count; Systemic Inflammation Response Index (SIRI): (Neutrophil count × Monocyte count)/Lymphocyte count; Neutrophil to Lymphocyte Ratio (NLR): Absolute neutrophil count/Absolute lymphocyte count; Lymphocyte to Monocyte Ratio (LMR): Lymphocyte count/Monocyte count; RDW to Platelet Ratio (RPR): Red blood cell distribution width/Platelet count; Mean Platelet Volume to Platelet Count Ratio (MPV/PC): Mean platelet volume/Platelet count; Lymphocyte-to-Monocyte Ratio (LMR): Lymphocyte count/Monocyte count; Neutrophil to Albumin Ratio (NPAR): Neutrophil percentage (%) × 100/Albumin concentration (g/dL); Alanine Aminotransferase to Aspartate Aminotransferase Ratio (AST/ALT): Alanine aminotransferase concentration/Aspartate aminotransferase concentration.

### 2.3 Statistical analysis

All statistical analyses were performed using SPSS for Windows version 27.0. For normally distributed data, they were presented as mean ± standard deviation (SD) and an independent samples *t*-test was used for comparisons between the two groups. For non-normally distributed data, they were presented as median and IQR and the Mann–Whitney *U* test was applied for comparisons between the two groups. For certain specified variables, Pearson correlation analysis was performed if the data followed a normal distribution, while Spearman correlation analysis was used if the data did not. Binary logistic regression analysis [odds ratio (OR), 95% confidence interval (CI)] was used to assess the relationship between each blood marker and the risk of SCP. A logistic regression model was used to generate receiver operating characteristic (ROC) curves. The overall discriminative ability was evaluated based on the area under the curve (AUC). An AUC >0.9 was considered excellent, AUC >0.8 was considered good, and AUC >0.7 was considered fair. A *p*-value of less than 0.05 was considered statistically significant.

## 3 Results

### 3.1 Clinical characteristics of participants

**Figure 1:**
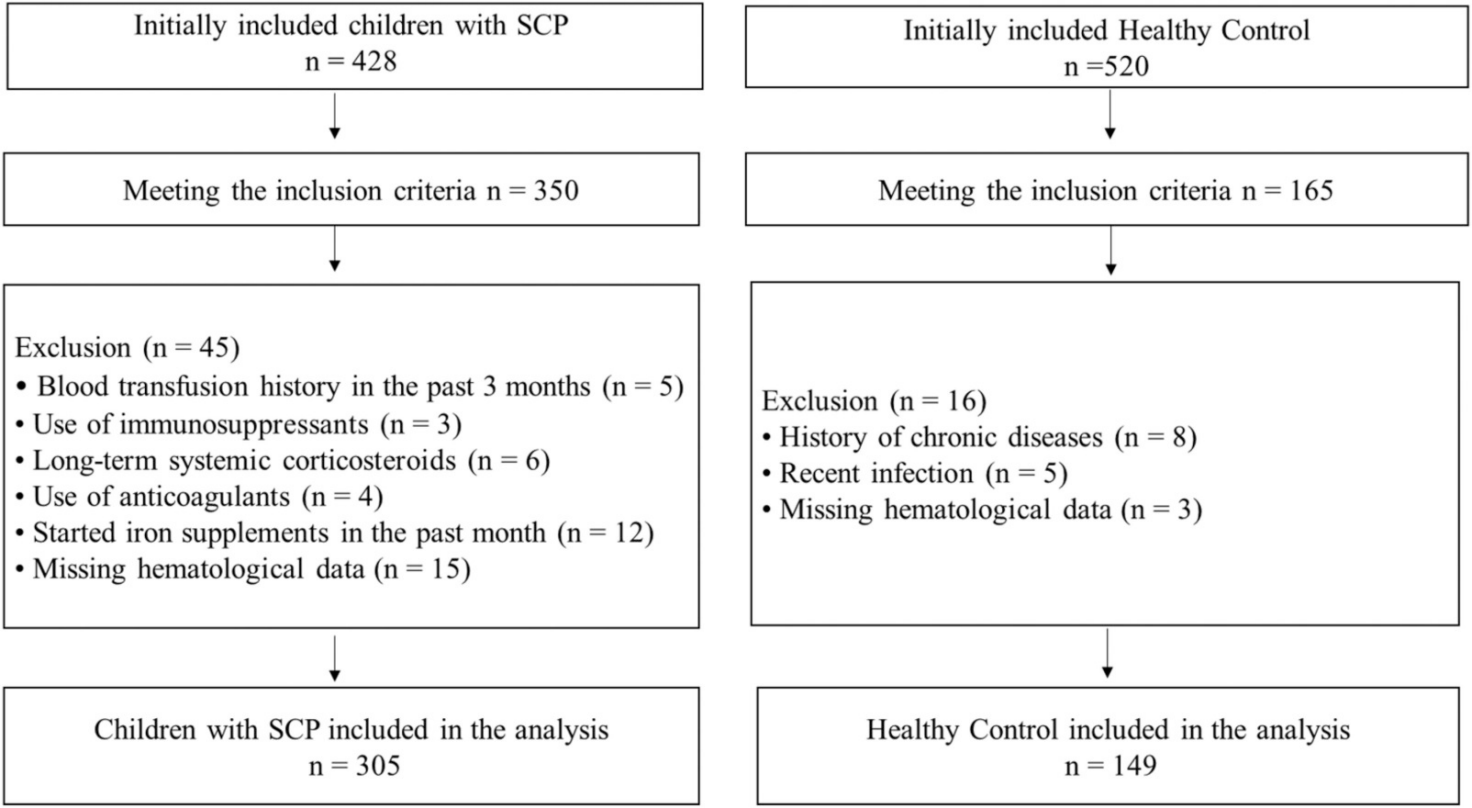
Flowchart of participant recruitment.

The average age of the 305 children in the SCP group was 7.10 ± 2.288 years. The average age of the 149 healthy children in the control group was 6.67 ± 2.705 years. There was no significant difference in age distribution between the SCP group and the control group (*χ*^2^ = 1.046, *p* = 0.306). Table 1 shows the age characteristics of all participants. The participant recruitment process is illustrated in Figure 1.

**Table 1:** Age characteristics of participants.

Characteristic	SCP (*n* = 305)	Healthy control (*n* = 149)	*χ* ^2^	*p*
Age (year), *n* (%)			1.046	0.306
3 ≤ *x* ≤ 6	140 (45.90%)	76 (51.01%)		
6 < *x* ≤ 12	165 (54.10%)	73 (48.99%)		

### 3.2 Blood marker comparison analysis

In our study, values for ALP, Cr, urea, UA, TB, AST/ALT, PLR, SII, SIRI, RPR, MPV/PC, NLR, NPAR, and LMR were collected or calculated. Table 2 shows the mean ± standard deviation of all data.

**Table 2:** Blood marker information of participants.

Biomarkers	SCP	CON	*p*
ALP (U/L)	215.10 (185.05–263.00)	261.00 (224.50–294.50)	<0.01
Cr (µmol/L)	37.40 (26.00–42.50)	39.00 (34.00–44.00)	0.025
Blood urea (mmol/L)	4.71 (3.81–5.51)	4.50 (3.79–5.37)	0.344
UA (µmol/L)	265.00 (183.60–305.85)	264.00 (231.50–307.00)	0.742
TB (g/L)	71.60 (67.55–75.50)	66.60 (64.15–70.25)	<0.001
AST/ALT (U/L)	0.63 (0.46–0.84)	0.49 (0.41–0.65)	<0.01
PLR	98.62 (75.93–129.09)	102.46 (79.30–123.05)	0.633
SII	339.12 (218.79–511.56)	279.76 (185.33–424.77)	0.004
SIRI	0.55 (0.35–0.95)	0.42 (0.29–0.71)	<0.01
RPR	0.46 (0.04–0.05)	0.45 (0.04–0.05)	0.972
MPV/PC	0.03 (0.02–0.04)	0.04 (0.03–0.04)	<0.01
NLR	1.20 (0.80–1.87)	0.99 (0.71–1.49)	0.001
NPAR	1.11 ± 0.30	1.04 ± 0.28	0.017
LMR	6.20 (4.25–8.50)	6.56 (4.93–8.18)	0.251

According to the statistical analysis, the NPAR data follows a normal distribution and was analyzed using the t-test, presented as mean ± standard deviation (SD). The NPAR levels in children with SCP were significantly lower than those in the healthy group (*p* < 0.05). Other data did not follow a normal distribution and were analyzed using non-parametric tests, presented as median and interquartile range (IQR). The levels of ALP, Cr, SII, and MPV/PC in the SCP group were significantly lower than those in the healthy control group (*p* < 0.05; Table 2). The levels of AST/ALT, NLR, SIRI, and TB in the SCP group were significantly higher than those in the healthy control group (*p* < 0.05). There were no significant differences in the levels of urea, uric acid, PLR, RPR, and LMR between the SCP group and the healthy control group.

### 3.3 Logistic regression analysis

**Figure 2:**
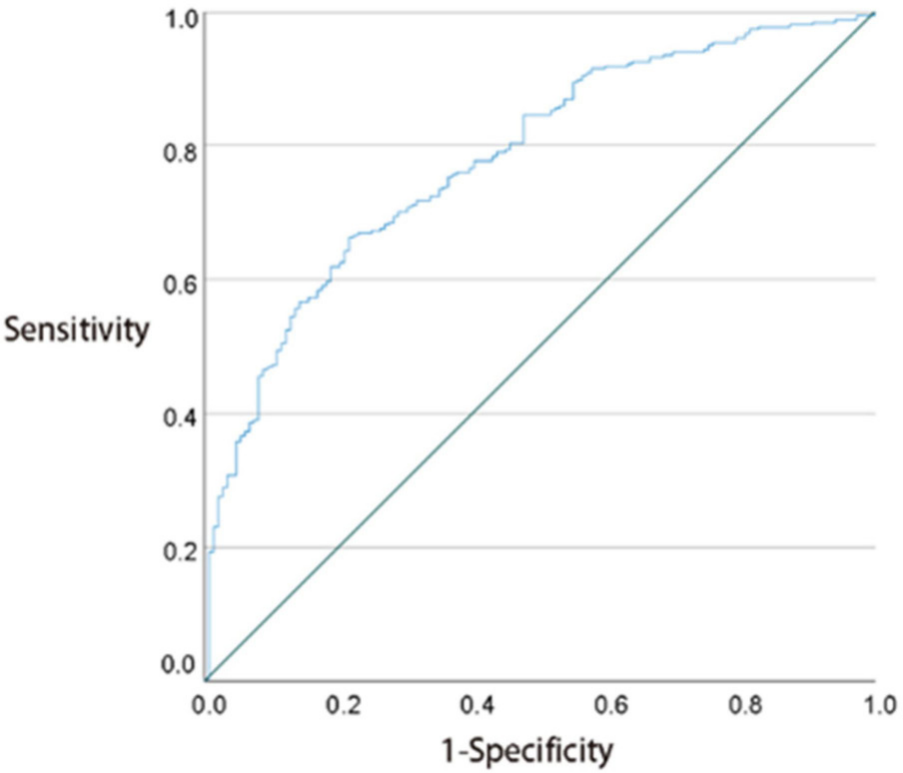
ROC curve of the combined indicators.

We identified NPAR, ALP, Cr, SII, SIRI, MPV/PC, AST/ALT, NLR, and TB as potential blood markers for distinguishing children with SCP from normal children. A multivariate binary logistic regression analysis using the “enter” variable selection strategy was conducted to study the impact of different variables on the risk of SCP. The results are shown in Table 3. It was found that ALP, Cr, SII, and MPV/PC were protective factors for SCP, while AST/ALT and NLR were risk factors for SCP. Since the *p*-values for some indicators were greater than 0.05, indicating no statistical significance in the multivariate model, total protein, as well as SIRI and NPAR indicators, were excluded. The recalculated results are shown in Table 4: ALP is a protective factor for SCP in China (OR = 0.991, 95% CI: 0.988–0.995, *p* < 0.001); Cr is a protective factor for SCP in China (OR = 0.964, 95% CI: 0.937–0.993, *p* = 0.013); SII is a protective factor for SCP in China (OR = 0.997,95% CI: 0.994–0.999, *p* = 0.006); AST/ALT increases the risk of SCP in China by 8.279 times (95% CI: 3.181–21.548, *p* < 0.001); MPV/PC is a very strong protective factor for SCP in China (OR = 0.000, 95% CI: 0.000–0.000, *p* < 0.001); NLR increases the risk of SCP in China by 3.548 times (95% CI: 1.601–7.860, *p* = 0.002). Subsequently, the effectiveness of these blood markers in diagnosing CP was evaluated through ROC curve analysis. The ROC curve indicates an AUC of 0.781, with a 95% confidence interval (CI) of 0.738–0.824, and *p* < 0.001. The ROC curve is presented in Figure 2.

**Table 3:** Results of logistic regression analysis.

Indicator	*B*	S.E.	*p*	Exp (B)	Upper confidence interval for Exp (B)	Lower confidence interval for Exp (B)
ALP	−0.009	0.002	<0.01	0.991	0.988	0.995
Cr	−0.034	0.015	0.024	0.967	0.939	0.996
TB	0.002	0.003	0.492	1.002	0.996	1.009
AST/ALT	2.163	0.490	<0.001	8.699	3.328	22.739
SII	−0.004	0.001	0.006	0.996	0.994	0.999
SIRI	0.634	0.375	0.091	1.885	0.904	3.930
MPV/PC	−37.211	10.037	<0.001	<0.001	<0.001	<0.001
NLR	0.947	0.452	0.036	2.578	1.064	6.249
NPAR	0.108	0.417	0.795	1.114	0.492	2.523

**Table 4:** Simplified logistic regression results.

Indicator	*B*	S.E.	p	Exp (B)	Upper confidence interval for Exp (B)	Lower confidence interval for Exp (B)
ALP	−0.009	0.002	<0.001	0.991	0.988	0.995
Cr	−0.036	0.015	0.013	0.964	0.937	0.993
AST/ALT	2.114	0.488	<0.001	8.279	3.181	21.548
SII	−0.003	0.001	0.006	0.997	0.994	0.999
MPV/PC	−36.115	9.647	<0.001	<0.001	<0.001	<0.001
NLR	1.266	0.406	0.002	3.548	1.601	7.860

### 3.4 Subgroup analysis

All children with SCP were divided into two age groups: preschool (3–6 years) with 140 participants and school-age (7–12 years) with 165 participants. The differences in ALP, Cr, AST/ALT, SII, MPV/PC, and NLR between the different age groups were compared.

According to the statistical analysis, the data does not follow a normal distribution and was analyzed using non-parametric tests, presented as median and interquartile range (IQR). In children with spastic cerebral palsy aged 3–6 years, the levels of Cr, AST/ALT, SII, MPV/PC, and NLR were significantly lower than those in children aged 7–12 years. There was no significant difference in ALP levels between the two age groups of children with SCP. The results are shown in Table 5. Correlation analysis showed that Cr, AST/ALT, SII, MPV/PC, and NLR were positively correlated with age (*p* < 0.05). The results are shown in Table 6.

**Table 5:** Blood marker information of SCP by Age group.

Age (year)	ALP	Cr	AST/ALT	SII	MPV/PC	NLR
3–6	215.00 (185.25–258.30)	33.85 (29.10–38.18)	0.55 (0.41–0.69)	289.17 (189.06–438.33)	0.03 (0.02–0.03)	0.95 (0.69–1.56)
7–12	217.00 (185.00–268.90)	39.90 (34.35–44.40)	0.73 (0.54–1.00)	378.00 (248.32–63.00)	0.03 (0.02–0.04)	1.33 (1.00–2.26)
p	0.615	<0.01	<0.01	<0.01	0.014	<0.01

**Table 6:** Correlation analysis of blood markers and Age.

Indicator	Correlation coefficient with age	*p*
Cr	0.425	<0.01
AST/ALT	0.365	<0.01
SII	0.262	<0.01
MPV/PC	0.143	0.012
NLR	0.335	<0.01

## 4 Discussion

In our study, we found that blood markers in children with SCP, including ALP, Cr, SII, and MPV/PC, are protective factors for SCP, while AST/ALT and NLR are risk factors for SCP. These six indicators may be of significant importance for monitoring the overall health status of children with spastic cerebral palsy aged 3–12 years.

The immune system plays a crucial role in brain homeostasis, resilience, and brain reserve, and the interaction between the immune system and the brain is gradually becoming a focus in the study of neurological diseases (12). Under normal physiological conditions, white blood cells have difficulty crossing the blood-brain barrier (BBB) and are primarily located near the brain's borders. On the other hand, peripheral cytokines can penetrate the brain via endothelial transport proteins, allowing them to affect brain function by regulating neurotransmitters and neural activity (13). In cases of chronic inflammation, the integrity of the blood-brain barrier may be compromised, promoting the entry of circulating cytokines and white blood cells into the brain. Once peripheral inflammatory signals enter the brain, they primarily reside in immune cells, leading to brain damage, typically manifested as chronic neuroinflammation and neurodegeneration (14). It is important to note that children with spastic cerebral palsy, due to poor posture control, scoliosis, and impaired oropharyngeal function, are more susceptible to acute lower respiratory tract infections and chronic lung diseases. Respiratory system diseases are one of the most common reasons for hospitalization in children with cerebral palsy (15, 16). This repeated infection state can lead to long-term chronic peripheral inflammation, further exacerbating blood-brain barrier damage and central nervous system inflammation, forming a vicious cycle.

Furthermore, the ratio of white blood cell counts, including NLR, PLR, LMR, and SII, has been proposed as effective measures to assess peripheral immune strength (17). Neutrophils and lymphocytes are important parameters of the inflammatory response, so NLR is considered an indicator reflecting systemic inflammation and physiological stress, representing the balance between innate and adaptive immunity (18). Multiple studies have shown that children with cerebral palsy have higher NLR levels compared to healthy individuals (19, 20). Similarly, in our study, the NLR level in children with spastic cerebral palsy in China was significantly higher. These findings suggest that spastic cerebral palsy is associated with a systemic inflammatory state. The increased NLR in children with cerebral palsy in this age group may be due to multiple factors: First, repeated respiratory infections can directly lead to an increase in neutrophil count and a relative decrease in lymphocytes (21). Second, long-term malnutrition may affect immune system function, such as vitamin D deficiency in children, which can cause elevated NLR levels (22). Additionally, some children with spastic cerebral palsy who also have epilepsy may have a poor response to AEDs, developing drug-resistant epilepsy, which leads to increased release of inflammatory mediators and further exacerbates central nervous system inflammation (23). Therefore, the observed increase in NLR in this study may be the result of a combination of repeated infections, malnutrition, and medication use, rather than solely a marker of neuroinflammation. NLR can serve as a reference indicator for assessing the systemic inflammatory state in Chinese children with spastic cerebral palsy.

SII is calculated based on the ratio of neutrophil count to lymphocyte count, multiplied by platelet count, and can also reflect systemic inflammation. A study in Turkey found that the SII level in children with cerebral palsy was significantly higher than that in healthy children (19). However, in our study, we found that the SII level in children with spastic cerebral palsy in China was lower than in healthy children. This may be because the platelet count in this group of children with spastic cerebral palsy was generally lower than that in healthy children, although still within the normal range. At the same time, in this study, the MPV/PC level in children with spastic cerebral palsy in China was also lower than that in healthy children, indicating that the mean platelet volume was generally lower within the normal range compared to healthy children. This difference suggests that there is heterogeneity in blood markers in different populations and in various forms of cerebral palsy. Both SII and MPV/PC were found to be protective factors for spastic cerebral palsy in this study, suggesting that appropriately promoting platelet count and increasing platelet volume may reduce the risk of the disease. The low platelet count may be related to the malnutrition commonly observed in children with cerebral palsy in this age group.

Healthy dietary fats help in hematopoiesis and maintain good cell membrane fluidity. The intake and regulation of fatty acids are particularly important for megakaryocyte maturation and increased platelet count. Adjusting dietary fatty acids, especially those rich in polyunsaturated fatty acids, could be a feasible strategy for regulating platelet count (24). Similarly, another study confirmed that a ketogenic diet significantly increases ketone body levels in the blood, along with an increase in platelet count. The ketogenic diet enhances circulating β-OHB levels, and ketone bodies enter megakaryocytes via the MCT1-dependent pathway (25). This process regulates the expression of platelet production genes through histone acetylation mechanisms, leading to increased platelet count. At the same time, nutritional management for children with cerebral palsy in this age group should focus on a balanced diet, particularly ensuring adequate intake of hematopoietic nutrients such as iron, folic acid, and vitamin B12, to improve their overall hematological status. For children with spastic cerebral palsy, regular monitoring of blood routine tests should be conducted, with attention to NLR, SII, and MPV/PC. Medication and dietary plans should be adjusted as needed.

In terms of metabolism, the liver is an essential organ for digestion, metabolism, and detoxification, typically assessed through biomarkers such as ALT, AST, ALP, bilirubin, proteins, and coagulation factors. Similarly, kidney function is crucial for waste excretion and electrolyte regulation, evaluated through biomarkers such as urea, Cr, UA, and electrolytes. Our study found that the AST/ALT levels in children with spastic cerebral palsy were higher than those in healthy children. This suggests that there may be underlying liver dysfunction in some cerebral palsy patients. These changes could be associated with the malnutrition commonly seen in CP patients, where dietary deficiencies impair liver function, as well as metabolic abnormalities caused by changes in protein metabolism (26). Additionally, AST is a marker of skeletal muscle injury (27), and the lower limb skeletal muscles in children with spastic cerebral palsy are often in a state of prolonged spasm, which may lead to elevated AST levels. In addition, several AEDs, such as sodium valproate, phenytoin, and carbamazepine, have potential hepatotoxicity, and long-term use can lead to elevated transaminase levels (28, 29). The elevated AST and ALT levels observed in the cerebral palsy group in this study may be the result of a combination of liver dysfunction due to malnutrition, skeletal muscle damage caused by spasticity, and the hepatotoxicity of AEDs. This finding suggests that clinicians should regularly monitor liver function indicators in the follow-up management of school-aged children with cerebral palsy. For children with a significantly increased AST/ALT ratio, clinical attention should be given, with close follow-up and early intervention.

This study also found that ALP and Cr levels in children with SCP in China were lower than those in healthy children, and these markers were identified as protective factors for SCP. This result differs from findings in cerebral palsy populations from other regions. The reasons may be related to the specific characteristics of the child population or the spastic subtype in China. ALP is associated with skeletal growth and bone metabolism (30), and the results of this study may suggest that higher ALP levels within a certain range indicate better skeletal development, thus lowering the probability of developing cerebral palsy. Additionally, studies have confirmed that higher Cr levels are associated with better muscle mass (31). Higher Cr levels within the normal range in adolescents are linked to higher bone mineral density (BMD), and Cr may be a potential biomarker for bone health in adolescents (32). Similarly, the results of this study may suggest that higher creatinine levels within a certain range indicate better musculoskeletal development, thus reducing the likelihood of cerebral palsy.

The age subgroup analysis showed that the levels of Cr, AST/ALT, SII, MPV/PC, and NLR increased with age in children with spastic cerebral palsy, reflecting the long-term progression of the disease and the multi-system pathophysiological burden. Therefore, early intervention in the clinical management of spastic cerebral palsy is essential to delay the progressive physical damage associated with aging.

This study is the first to systematically analyze the characteristics of routine hematological indicators, covering inflammation, metabolism, and platelet function, in Chinese children with spastic cerebral palsy. In clinical practice, for the health management of children with spastic cerebral palsy aged 3–12 years, multiple hematological indicators should be systematically monitored, combined with the child's medical history, nutritional assessment, medication usage, and infection history for comprehensive analysis, to achieve personalized treatment and management.

This study has some limitations. First, it only included children aged 3–12 years, and did not cover infants and toddlers aged 0–3 years with spastic cerebral palsy. The main reason for excluding the 0–3 age group is that hematological parameters in infancy and early childhood undergo rapid developmental changes, and these values differ significantly from those of other age groups (33). However, the lack of data for the 0–3 age group somewhat limits the generalization of the findings to younger children. Future prospective studies should include infants and toddlers aged 0–3 years to fully understand the dynamic evolution of hematological parameters in children with spastic cerebral palsy. Second, as this study was retrospective in design, the medical records did not systematically include standardized functional assessment data such as the Gross Motor Function Classification System (GMFCS) and the Eating and Drinking Ability Classification System (EDACS). The GMFCS classification directly reflects the degree of motor function limitation, and children with different GMFCS levels have significant differences in physical activity, feeding ability, and nutritional status, all of which may affect hematological parameters. The lack of GMFCS data prevented subgroup analysis based on disease severity, making it impossible to explore the relationship between functional limitations and hematological abnormalities. Future research should include standardized functional assessment tools like GMFCS and EDACS, and conduct stratified analysis of children with cerebral palsy based on different functional levels.

## 5 Conclusion

This study developed a comprehensive assessment model based on six routine hematological indicators (alkaline phosphatase, creatinine, AST/ALT, SII, MPV/PC, NLR) covering metabolic function, inflammatory status, and platelet function. The model's ROC curve AUC value reached 0.781 and can serve as a reference tool for evaluating the overall health status of Chinese children aged 3–12 years with spastic cerebral palsy. Since the selected indicators are derived from routine blood tests commonly conducted in primary healthcare institutions, the model has the advantages of being cost-effective, convenient, and easy to promote. It provides a new strategy for clinicians to continuously monitor the health status of children with spastic cerebral palsy, detect complications early, and optimize individualized treatment plans.

## Data Availability

The raw data supporting the conclusions of this article will be made available by the authors, without undue reservation.

## References

[1] Novak I Jackman M Finch-Edmondson M Fahey M. Cerebral palsy. Lancet. (2025) 406(10499):174–88. 10.1016/S0140-6736(25)00686-5 40550230

[2] Zhou H Peng T Wei M Zhang J Zhao Y Le W Validity and predictability of mid-upper arm circumference for nutrition screening in outpatient preschoolers with cerebral palsy. Front Nutr. (2025) 12:1609032. 10.3389/fnut.2025.1609032 40977989 PMC12447903

[3] Zagierski M Górska A Zagierska A Augustyńska J Seweryn M Szlagatys-Sidorkiewicz A. Home enteral nutrition in patients with cerebral palsy in the years 2012–2022: a longitudinal analysis of data from the national health fund of Poland. Nutrients. (2024) 16(15):2394. 10.3390/nu16152394 39125275 PMC11314170

[4] Feroze N Karim T Ostojic K McIntyre S Barnes EH Lee BC Clinical features associated with epilepsy occurrence, resolution, and drug resistance in children with cerebral palsy: a population-based study. Dev Med Child Neurol. (2024) 66(6):793–803. 10.1111/dmcn.15807 38059324

[5] Sanner JR Jain K Williams J Hurley MN. Antibiotics for chronic pulmonary infection in children with a neurodisability (neurodevelopmental disorder). Cochrane Database Syst Rev. (2023) 2(2):CD013813. 10.1002/14651858.CD013813.pub2 36757320 PMC9909774

[6] Zareen Z Strickland T Fallah L McEneaney V Kelly L McDonald D Cytokine dysregulation in children with cerebral palsy. Dev Med Child Neurol. (2021) 63(4):407–12. 10.1111/dmcn.14724 33185287

[7] Jiang NM Cowan M Moonah SN Petri WA Jr. The impact of systemic inflammation on neurodevelopment. Trends Mol Med. (2018) 24(9):794–804. 10.1016/j.molmed.2018.06.008 30006148 PMC6110951

[8] Oyarzábal A Musokhranova U Barros LF García-Cazorla A. Energy metabolism in childhood neurodevelopmental disorders. EBioMedicine. (2021) 69:103474. 10.1016/j.ebiom.2021.103474 34256347 PMC8324816

[9] Tezol Ö Yalçın SS Reçber T Yirün A Balcı Özyurt A Okuyaz Ç Metabolomics analysis of children with spastic cerebral palsy: a case-control study. BMC Pediatr. (2025) 25(1):494. 10.1186/s12887-025-05828-w 40596988 PMC12220464

[10] Su H Luo J Han M Zhang Y Hou Q Fan D The relationship between functional status and hematological parameters in children with spastic cerebral palsy: a retrospective cross-sectional study. Transl Pediatr. (2025) 14(6):1087–102. 10.21037/tp-2024-564 40688203 PMC12268867

[11] Xu L. Cerebral Palsy. Beijing: People’s Medical Publishing House (2018). p. 483.

[12] Zhong X Qiang Y Wang L Zhang Y Li J Feng J Peripheral immunity and risk of incident brain disorders: a prospective cohort study of 161,968 participants. Transl Psychiatry. (2023) 13(1):382. 10.1038/s41398-023-02683-0 38071240 PMC10710500

[13] Banks WA. Blood-brain barrier transport of cytokines: a mechanism for neuropathology. Curr Pharm Des. (2005) 11(8):973–84. 10.2174/1381612053381684 15777248

[14] Delpech JC Madore C Nadjar A Joffre C Wohleb ES Layé S. Microglia in neuronal plasticity: influence of stress. Neuropharmacology. (2015) 96(Pt A):19–28. 10.1016/j.neuropharm.2014.12.034 25582288

[15] Proesmans M Vermeulen F Boon M. Understanding and managing respiratory infections in children and young adults with neurological impairment. Expert Rev Respir Med. (2023) 17(3):203–11. 10.1080/17476348.2023.2192483 36932917

[16] Reid SM Vendeleur M Wurzel D Frayman K Osowicki J Crompton K Respiratory admissions and impact of COVID-19 lockdowns for children with severe cerebral palsy. Dev Med Child Neurol. (2025) 67(12):1582–9. 10.1111/dmcn.16346 40344424 PMC12618963

[17] Nøst TH Alcala K Urbarova I Byrne KS Guida F Sandanger TM Systemic inflammation markers and cancer incidence in the UK biobank. Eur J Epidemiol. (2021) 36(8):841–8. 10.1007/s10654-021-00752-6 34036468 PMC8416852

[18] Paolini M Harrington Y Raffaelli L Poletti S Zanardi R Colombo C Neutrophil to lymphocyte ratio and antidepressant treatment response in patients with major depressive disorder: effect of sex and hippocampal volume. Eur Neuropsychopharmacol. (2023) 76:52–60. 10.1016/j.euroneuro.2023.07.010 37544076

[19] Orhan O Gokdemir GS. Assessment of iron metabolism and inflammation in children with cerebral palsy. J Clin Med. (2024) 14(1):61. 10.3390/jcm14010061 39797144 PMC11721373

[20] Riewruja K Amarase C Osateerakun P Weerasopone S Limpaphayom N Honsawek S. Neutrophil-to-lymphocyte ratio predicts the severity of motor impairment in cerebral palsy children living at home and the rehabilitation center: a comparative study. Biomed Rep. (2020) 13(6):63. 10.3892/br.2020.1370 33149907 PMC7605123

[21] Wittermans E van de Garde EM Voorn GP Aldenkamp AF Janssen R Grutters JC Neutrophil count, lymphocyte count and neutrophil-to-lymphocyte ratio in relation to response to adjunctive dexamethasone treatment in community-acquired pneumonia. Eur J Intern Med. (2022) 96:102–8. 10.1016/j.ejim.2021.10.030 34782191

[22] Okuyan O Dumur S Elgormus N Uzun H. The relationship between vitamin D, inflammatory markers, and insulin resistance in children. Nutrients. (2024) 16(17):3005. 10.3390/nu16173005 39275320 PMC11396811

[23] Rashdan AR El-Haggar SM Kishk AM Mostafa TM. Probiotic supplementation as an adjuvant therapy in pediatric drug-resistant epilepsy: a double-blind placebo-controlled trial. Pharmacotherapy. (2026) 46(3):e70117. 10.1002/phar.70117 41693686

[24] Barrachina MN Pernes G Becker IC Allaeys I Hirsch TI Groeneveld DJ Efficient megakaryopoiesis and platelet production require phospholipid remodeling and PUFA uptake through CD36. Nat Cardiovasc Res. (2023) 2(8):746–63. 10.1038/s44161-023-00305-y 39195958 PMC11909960

[25] Xie S Jiang C Wu M Ye Y Wu B Sun X Dietary ketone body-escalated histone acetylation in megakaryocytes alleviates chemotherapy-induced thrombocytopenia. Sci Transl Med. (2022) 14(673):eabn9061. 10.1126/scitranslmed.abn9061 36449600

[26] Caramico-Favero DCO Guedes ZCF Morais MB. Food intake, nutritional status and gastrointestinal symptoms in children with cerebral palsy. Arq Gastroenterol. (2018) 55(4):352–7. 10.1590/s0004-2803.201800000-78 30785518

[27] Lian R Liu Q Jiang G Zhang X Tang H Lu J Blood biomarkers for sarcopenia: a systematic review and meta-analysis of diagnostic test accuracy studies. Ageing Res Rev. (2024) 93:102148. 10.1016/j.arr.2023.102148 38036104

[28] Chalasani N Bonkovsky HL Stine JG Gu J Barnhart H Jacobsen E Clinical characteristics of antiepileptic-induced liver injury in patients from the DILIN prospective study. J Hepatol. (2022) 76(4):832–40. 10.1016/j.jhep.2021.12.013 34953957 PMC8944173

[29] Pal R Singh K Khan SA Chawla P Kumar B Akhtar MJ. Reactive metabolites of the anticonvulsant drugs and approaches to minimize the adverse drug reaction. Eur J Med Chem. (2021) 226:113890. 10.1016/j.ejmech.2021.113890 34628237

[30] Cannalire G Pilloni S Esposito S Biasucci G Franco D Street A Alkaline phosphatase in clinical practice in childhood: focus on rickets. Front Endocrinol (Lausanne). (2023) 14:1111445. 10.3389/fendo.2023.1111445 36817604 PMC9931734

[31] Groothof D Shehab NBN Erler NS Post A Kremer D Polinder-Bos HA Creatinine, cystatin C, muscle mass, and mortality: findings from a primary and replication population-based cohort. J Cachexia Sarcopenia Muscle. (2024) 15(4):1528–38. 10.1002/jcsm.13511 38898741 PMC11294032

[32] Fang J Kong G Wang Y Pan K. Association between serum creatinine level within normal range and bone mineral density in adolescents. Arch Pediatr. (2022) 29(5):364–9. 10.1016/j.arcped.2022.05.002 35637044

[33] Song W Yan R Peng M Jiang H Li G Cao S Age and sex specific reference intervals of 13 hematological analytes in Chinese children and adolescents aged from 28 days up to 20 years: the PRINCE study. Clin Chem Lab Med. (2022) 60(8):1250–60. 10.1515/cclm-2022-0304 35607280

